# Co-Delivery of Ferrostatin‑1 and M2 Macrophage-Derived Exosomal Signals via Engineered Hybrid Nanovesicles Enables Synergistic Neuroprotection in Traumatic Brain Injury

**DOI:** 10.1021/acsami.6c01290

**Published:** 2026-04-07

**Authors:** Wenyan Hao, Nan Sun, Ruifen Xue, Junkai Chang, Xiaocong Pang, Ying Zhou, Chunsheng Gao

**Affiliations:** † Department of Pharmacy, 26447 Peking University First Hospital, Xishiku Street, Xicheng District, Beijing 100034, China; ‡ State Key Laboratory of Toxicology and Medical Countermeasures, 96705 Beijing Institute of Pharmacology and Toxicology, Beijing 100085, China; § State Key Laboratory of Advanced Drug Delivery and Release Systems, Shandong Luye Pharmaceutical Co., Ltd., Yantai, Shandong 264003, PR China; ∥ 4919 University College London, Flat 21, Dickens House, London WC1E 6BT, U.K.

**Keywords:** traumatic brain injury (TBI), ferroptosis inhibition, immune reprogramming, biomimetic nanovesicle, neuroinflammation modulation

## Abstract

Secondary brain injury after traumatic brain injury (TBI) is driven largely by ferroptosis-induced neuronal death and maladaptive neuroinflammation. Current therapies are limited by poor drug delivery and the narrow scope of single-pathway interventions. Here, we report a biomimetic hybrid nanovesicle (hMLV) engineered to codeliver the ferroptosis inhibitor ferrostatin-1 (Fer-1) and M2 macrophage–derived exosomes, enabling simultaneous suppression of neuronal ferroptosis and reprogramming of the immune microenvironment. The liposomal core encapsulates hydrophobic Fer-1 to enhance solubility and stability, while the exosomal membrane promotes blood–brain barrier penetration, lesion targeting via chemokine receptors, and immune evasion through CD47 expression. Within injured brain tissue, released Fer-1 restores glutathione peroxidase 4 (GPX4) activity, reduces lipid peroxidation, and prevents ferroptotic neuronal death. Concurrently, exosomal cytokines such as interleukin-10 and transforming growth factor-β drive macrophage polarization toward a reparative M2 phenotype, mitigating neuroinflammation. This dual mechanism establishes a positive therapeutic cycle: ferroptosis inhibition dampens inflammatory triggers, while M2 polarization reduces oxidative stress. In a murine TBI model, hMLV treatment conferred superior neuroprotection and functional recovery compared with monotherapies. These findings highlight hMLV as a clinically translatable nanoplatform for synergistic, mechanism-guided intervention in secondary brain injury.

## Graphical abstract

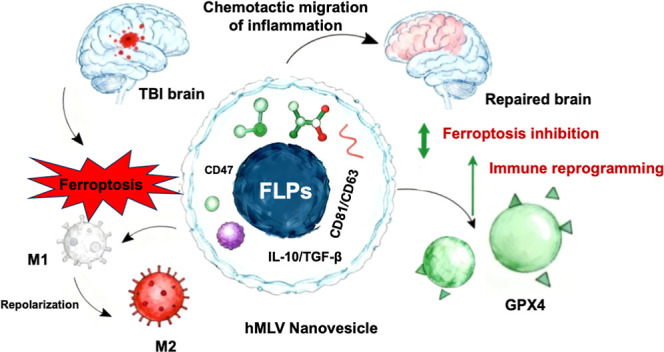

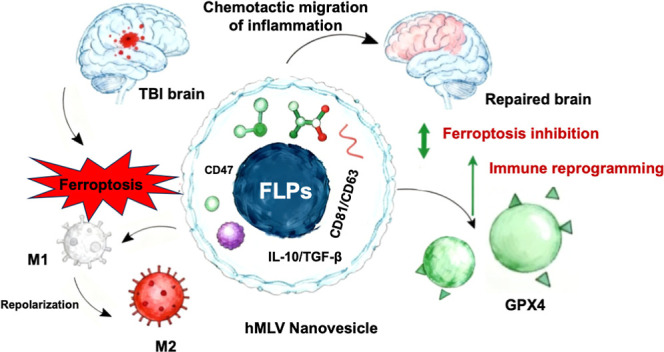

## 1 Introduction

Traumatic brain injury (TBI) represents a major global health challenge and is a leading cause of death and long-term disability worldwide. It is estimated that approximately 10 million people are hospitalized or die each year as a direct result of TBI.^1–3^ The profound social and economic burden of TBI underscores the urgent need for effective therapeutic strategies; however, current treatment options remain extremely limited. The pathological cascade of TBI comprises the primary mechanical insult and a more insidious secondary injury phase. The latter progresses over hours to months following the initial trauma and is driven by complex biochemical, cellular, and molecular processes triggered by the primary injury.^4,5^ Importantly, this secondary phase, characterized by progressive neuronal loss and neuroinflammation, accounts for much of the long-term functional impairment associated with TBI and represents a critical therapeutic window.^6,7^

The core pathophysiological mechanisms of secondary injury involve two interconnected processes: dysregulated neuronal iron metabolism culminating in ferroptosis, and maladaptive immune responses driven by microglial and macrophage polarization. Ferroptosis is an iron-dependent, regulated form of cell death that is closely associated with neuronal loss following traumatic brain injury (TBI).^8,9^ This process is initiated by excessive presynaptic glutamate release, which overwhelms the cystine/glutamate antiporter system (system xc−). The resulting inhibition of cystine uptake depletes the precursor required for glutathione (GSH) synthesis.^10^ GSH deficiency subsequently leads to inactivation of glutathione peroxidase 4 (GPX4), a key enzyme responsible for reducing lipid peroxides within cellular membranes.^11^ Consequently, lipid reactive oxygen species (ROS) accumulate, driving iron-dependent peroxidation and neuronal ferroptosis.^12,13^ Concurrently, the brain’s immune microenvironment undergoes substantial pathological changes. Microglia and infiltrating macrophages predominantly polarize toward the proinflammatory M1 phenotype, releasing cytokines that exacerbate neuronal injury and tissue damage.^14–16^ In contrast, polarization toward the reparative M2 phenotype is suppressed; under physiological conditions, M2 cells secrete anti-inflammatory mediators such as interleukin-10 (IL-10) and promote tissue repair.^17–19^ This pathological shift toward M1 polarization creates a detrimental neuroinflammatory state that acts in concert with ferroptotic pathways to aggravate secondary injury.^20,21^

Although interventions targeting either pathway show therapeutic potential, their significant limitations have impeded clinical translation.^22,23^ Pharmacological inhibition of ferroptosis with agents such as ferrostatin-1 (Fer-1) has demonstrated neuroprotective effects in experimental models, including reduced iron deposition, attenuation of neuronal degeneration, and improved functional outcomes.^24–26^ However, Fer-1 is characterized by poor aqueous solubility, limited stability in vivo, and potential systemic toxicity. Most importantly, despite disruption of the blood–brain barrier, Fer-1 cannot be delivered to the injured parenchyma with sufficient efficiency or specificity, severely restricting its clinical utility.^27–29^ Likewise, strategies designed to restore the balance of microglial/macrophage polarization, such as adoptive transfer of mesenchymal stromal cells or regulatory T cells, have exhibited anti-inflammatory effects. Nonetheless, these approaches face substantial challenges, including low homing efficiency of infused cells to the brain (with many retained in pulmonary capillaries), complex manufacturing processes, safety concerns, and variable therapeutic efficacy.^30,31^

Here, we present an integrated nanotherapeutic strategy designed to simultaneously inhibit neuronal ferroptosis and reprogram the immune microenvironment after brain injury. We engineered a biomimetic hybrid nanovesicle (hMLV), consisting of a liposome–exosome hybrid derived from reparative M2 macrophages and loaded with ferrostatin-1 (Fer-1). This design combines the complementary advantages of synthetic liposomes and natural exosomes. The liposomal core encapsulates hydrophobic Fer-1, enhancing its solubility, stability, and biocompatibility while enabling controlled release.^32^ The exosomal membrane shell confers critical biological functions: (i) enhanced brain targeting and penetration through exosomal tropism and chemokine receptor–mediated homing to inflamed lesions;^33,34^ (ii) immune evasion and prolonged circulation mediated by CD47-dependent inhibition of phagocytosis;^35^ and (iii) intrinsic delivery of immunoregulatory cargo, including anti-inflammatory cytokines (e.g., IL-10, TGF-β) and neurotrophic factors (e.g., M-CSF), which promote polarization of immune cells toward a reparative M2 phenotype.^36,37^

After accumulation in TBI lesions, hMLV exerts dual therapeutic effects. Sustained release of Fer-1 directly inhibits ferroptosis by reducing glutamate excitotoxicity, preserving GPX4 activity through maintenance of the system xc––GSH axis, and preventing lipid peroxidation. At the same time, M2-derived exosomal signals, including both membrane-associated molecules and soluble factors (e.g., IL-18), reprogram the local immune microenvironment by limiting proinflammatory M1 activity and supporting anti-inflammatory M2 responses. Notably, these mechanisms reinforce each other: inhibition of ferroptosis reduces one of the main drivers of neuroinflammation, while suppression of neuroinflammation decreases oxidative stress and dampens ferroptotic amplification. Together, these effects establish a positive feedback loop. By harnessing endogenous inflammatory homing signals and applying biomimetic nanotechnology, this platform addresses the delivery and efficacy barriers of previous monotherapies, offering a promising approach for reducing secondary brain injury and improving functional recovery following TBI.

## 2 Materials and Methods

### 2.1 Materials

Ferrostatin-1 (Fer-1) was purchased from Shanghai Aladdin Biochemical Technology Co., Ltd. (China). Lecithin was obtained from Shanghai Macklin Biochemical Co., Ltd. (China). Cholesterol and DSPE-mPEG2000 were purchased from Shanghai Aiweituo Biotechnology Co., Ltd. (China). DCFH-DA and BODIPY C11–581/591 were obtained from Absin Bioscience, Inc. (Shanghai, China). ELISA kits for GPX4, GSH, and IL-1β were purchased from Absin Bioscience, Inc. (Shanghai, China). All other chemicals were supplied by Sigma-Aldrich and were of analytical reagent grade unless otherwise specified.

### 2.2 Cell Culture and Animal Experiments

HT22, RBMVECs, and RAW264.7 cells were obtained from the Cell Resource Center of IBMS (Beijing, China). Cells were cultured in Dulbecco’s modified Eagle’s medium (DMEM) supplemented with 10% fetal bovine serum (FBS; Gibco) and 100 IU/mL penicillin, and maintained in a humidified incubator at 37 °C with 5% CO_2_. Male ICR mice (28–32 g) were supplied by SPF Biotechnology Co., Ltd. (Beijing, China). Animals were housed at 25 ± 1 °C under 50–60% relative humidity with free access to food and water.

### 2.3 Preparation and Characterization of hMLV

Liposomes were synthesized using the thin-film hydration method. Briefly, Fer-1, cholesterol, DSPE-mPEG2000, and soy lecithin were dissolved in dichloromethane and transferred to a round-bottom flask. The solvent was removed under reduced pressure with a rotary evaporator to form a thin lipid film, which was subsequently hydrated with ultrapure water for 30 min. The resulting suspension was sonicated at 60 W for 2 min at 4 °C and filtered through a 0.22 μm membrane. Particle size was measured, and liposomes were stored at 4 °C for further use.

RAW264.7 cells were cultured until reaching >80% confluence and then induced toward the M2 phenotype. Cells were treated with IL-4 (20 ng/mL) and IL-13 (20 ng/mL) for 48 h, and morphological changes were observed. After induction, cells were collected and analyzed for M2 macrophage markers (CD206, Arg-1) by flow cytometry.^38,39^ M2 macrophage-derived extracellular vesicles (M2-EVs) were purified by differential centrifugation. Conditioned medium was centrifuged at 2500*g* for 15 min to remove cells and debris, followed by centrifugation at 20,000*g* for 20 min. The exosome fraction was collected and ultracentrifuged at 150,000*g* for 100 min. The supernatant was discarded, and the pellet was resuspended in phosphate-buffered saline (PBS), frozen in liquid nitrogen, and stored at −80 °C for up to 1 week. Hybrid vesicles (hMLV) were generated by fusing M2-EVs with Fer-1-loaded liposomes (FLPs) as previously described.^40^ The particle size and zeta potential of the formulations were measured using dynamic light scattering (DLS), and morphology was characterized by transmission electron microscopy. To assess stability, hMLVs were stored in 1 × PBS or PBS containing 10% FBS at 37 °C. Particle size and zeta potential were measured over 72 h.

### 2.4 Drug Release Assay

Fer-1 formulations (1 mL) were placed into dialysis bags (MWCO 8000) and immersed in 50 mL PBS (pH 7.4). The system was maintained in a water bath at 37 °C with horizontal shaking (100 rpm) for 36 h. At predetermined intervals, 100 μL of release medium outside the dialysis bag was withdrawn and replaced with an equal volume of preheated PBS. Fer-1 concentrations were quantified using HPLC (Agilent 1200, USA), and cumulative release profiles were plotted.

### 2.5 Drug Loading and Encapsulation Efficiency

Unencapsulated Fer-1 was separated by ultracentrifugation (6000 rpm, 10 min) and quantified by HPLC in triplicate.^41^ HPLC analysis was performed on a C18 column (75 × 4.6 mm, 3.5 μm, Agilent) with a mobile phase of acetonitrile/water (0.1% formic acid) using a linear gradient (50:50 to 95:5). The injection volume was 20 μL, flow rate 1.0 mL/min, and detection wavelength 254 nm. The drug loading efficiency (LE) and encapsulation efficiency (EE) of hMLV were calculated using the following formulas: LE = MDTX-loaded/MhMLV, EE = MFer-1-loaded/M Fer-1-initial. MDTX-initial is the initial mass of Fer-1 used for hMLV preparation. M Fer-1-loaded is the mass of Fer-1 loaded in hMLV, which was determined by subtracting the amount of Fer-1 in the supernatant from M Fer-1-initial.

### 2.6 Western Blotting

Total protein was extracted and separated by SDS-PAGE, then transferred to PVDF membranes (0.22 μm). Membranes were blocked with 3% BSA for 1 h, incubated with primary antibodies at 4 °C overnight, and washed with TBST.^42^ After incubation with HRP-conjugated secondary antibodies (1:5000, 50 min), membranes were developed using ECL substrate. Band intensities were quantified with Quantity One v4.6.2 (Bio-Rad).

### 2.7 In Vitro BBB Permeability

An in vitro blood–brain barrier (BBB) injury model was established to evaluate the ability of the biomimetic nanovesicle hMLV to cross the BBB and target neurons under conditions mimicking traumatic brain injury.^43^ The model was constructed using a Transwell coculture system incorporating mouse brain microvascular endothelial cells (RBMVECs) and HT22 hippocampal neurons, recapitulating the physiological architecture of an intact BBB. To simulate the inflammatory microenvironment following TBI, the experimental group was treated with fMLP (100 nM) for 24 h to induce endothelial barrier dysfunction, while the control group remained untreated (Figure 3A). fMLP, a well-established inducer in vitro inflammation models, is widely used in studies of neuroinflammation, immune chemotaxis, and drug delivery across biological barriers due to its rapid action, dose controllability, and high reproducibility.

To validate the efficacy of the model, transendothelial electrical resistance (TEER) was measured to assess barrier integrity. Following fMLP stimulation, TEER values decreased by approximately 40% compared to baseline levels, confirming significant disruption of barrier integrity and successfully recapitulating the increased BBB permeability observed after TBI. Subsequently, free DiD, DiI-labeled M2-EVs (DiI-M2-EVs), or DiI-labeled hMLVs (DiI-hMLVs) were added to the upper chamber and coincubated for 48 h. After fixation and staining, cellular uptake was evaluated by confocal laser scanning microscopy (CLSM). Furthermore, HT22 cells were seeded at a density of 1 × 10^5^ cells/mL in 6-well plates and treated with the respective DiD-labeled formulations. After 48 h of incubation, cellular uptake was quantified by flow cytometry.

### 2.8 Traumatic Brain Injury Model

TBI was induced in ICR mice using the controlled cortical impact (CCI) model.^44^ Mice were anesthetized (50 mg/kg, i.p.) and analgesia was provided with 0.25% bupivacaine. After craniotomy (2 mm diameter, 2 mm lateral and posterior to bregma), impact was delivered (velocity 4.0 m/s, depth 1.5 mm, dwell time 0.2 s). Wounds were sutured and disinfected, and mice were monitored postsurgery. MRI was performed 2 days post-CCI to confirm modeling success.

### 2.9 In Vivo Brain Distribution

Mice were injected intravenously with saline, DiR-labeled FLPs, M2-EVs, or hMLVs (200 μL). In vivo fluorescence imaging was performed under isoflurane anesthesia at 6, 12, 24, and 48 h. At 24 h, mice were sacrificed, and major organs­(brain, heart, liver, spleen, lung, kidney)­were excised for ex vivo imaging.

### 2.10 Cell Viability Assay (CCK-8)

HT22 cells were seeded in 96-well plates (5000 cells/well). After 24 h, cells were treated with 1600 μM H_2_O_2_ and 10 μM RSL3 for 4 h, followed by exposure to different formulations for 24 h.^45^ Cell viability was assessed using CCK-8 (10 μL/well, 2 h incubation). Absorbance was measured at 450 nm. The cell viability was calculated using the following formula: Cell viability (%) = ((As – Ab)/(Ac – Ab)) × 100%, where As represents the experimental group, Ab represents the blank group, and Ac represents the control group.

### 2.11 Cell Apoptosis Assay

Cell apoptosis was evaluated using Annexin V-FITC/PI staining followed by flow cytometry. Briefly, 5 × 10^4^ −1 × 10^5^ cells were harvested, washed twice with PBS, and resuspended in 195 μL binding buffer. Annexin V-FITC (5 μL) and PI (10 μL) were added, and the samples were incubated at room temperature in the dark for 10–20 min prior to analysis.

### 2.12 GPX4 Activity Assay

HT22 cells (5 × 10^5^) were seeded in six-well plates and incubated for 24 h at 37 °C. Cells were treated with FLPs, M2-EVs, or hMLVs for 5 h, then washed with PBS and cultured for an additional 12 h under hypoxic conditions. GPX4 activity was quantified using a commercial glutathione peroxidase assay kit according to the manufacturer’s instructions (*n* = 3).

### 2.13 GSH Activity Assay

HT22 cells were seeded in six-well plates at a density of 4 × 10^5^ cells/well. Upon reaching 70–80% confluence, cells were treated with hMLVs for 12 h, collected, and homogenized on ice. GSH content was determined using a glutathione assay kit, following the manufacturer’s protocol. Untreated cells served as the baseline (100%). FLPs and M2-EVs were used as controls.

### 2.14 Flow Cytometry for Macrophage Polarization

RAW264.7 cells were plated at 1 × 10^6^ cells/well and polarized to the M2 phenotype by incubation with IL-4 and IL-13 (20 ng/mL each) for 24 h. Cells were then treated with Fer-1, M2-EVs, FLPs, or hMLVs for 24 h. Harvested cells (>5 × 10^5^/mL) were washed, resuspended in FACS buffer, and stained with anti-CD80, anti-CD163, or anti-CD206 antibodies at 4 °C in the dark. After fixation in 1% paraformaldehyde, at least 2 × 10^4^ cells per sample were analyzed by flow cytometry (BD Biosciences, USA). Data were processed with FlowJo software.

### 2.15 mNSS Scoring

Neurological function in CCI mice was evaluated using the modified Neurological Severity Score (mNSS), which assesses motor, reflex, sensory, and balance functions. Scores range from 0 (normal) to 18 (maximum deficit), with higher scores indicating more severe injury.

### 2.16 Morris Water Maze

Spatial learning and memory were assessed using the Morris water maze (MWM).^46^ Mice were trained for 5 consecutive days in the place navigation test, followed by a probe trial on day 6 in which the platform was removed. Behavioral performance was recorded with a video tracking system (AnyMaze 6.32, Stoelting, USA).

### 2.17 TUNEL Assay

Mice were euthanized 24 h after CCI, and brains were fixed in 4% paraformaldehyde, embedded in paraffin, and sectioned (4 μm). Sections were deparaffinized, rehydrated, subjected to antigen retrieval, and treated with protease and 3% hydrogen peroxide. Apoptotic cells were detected using a TUNEL staining kit with DAB as chromogen and hematoxylin counterstain. Sections were dehydrated, mounted, and imaged under a light microscope.

### 2.18 Nissl Staining

Paraffin-embedded brain sections were deparaffinized, hydrated, and stained with methylene blue Nissl solution. After differentiation, dehydration, and mounting, neuronal morphology was examined under a light microscope.^47^

### 2.19 In Vivo Evaluation of the Synergistic Mechanism of Ferroptosis-Immunotherapy

To assess the synergistic effects of ferroptosis-immunotherapy, CCI mice were randomly divided into four groups (*n* = 6) and intravenously injected with PBS (control), FLPs, M2-EVs, or hMLVs (2 mg/kg, three times per week). After 14 days, brain tissues were harvested, homogenized, and the supernatant was collected. GPX4 and GSH levels were quantified using ELISA. Neurons were stained with APC-conjugated anti-CD163 and PE-conjugated anti-CD206 antibodies, and the proportions of CD163-and CD206-positive cells were assessed by immunofluorescence and immunohistochemistry. Levels of IL-1β and IL-18 in damaged neuronal tissues were also measured by ELISA.

### 2.20 Toxicity Test

To evaluate potential toxicity, healthy mice were randomly divided into three groups and intravenously injected with PBS, FLPs, or hMLVs (200 μL, every 2 days for 7 days). Body weight was monitored daily. On the final day, ∼1.0 mL of blood was collected from the orbital sinus, centrifuged (4000 rpm, 10 min, 4 °C), and serum was analyzed using a biochemical analyzer. An additional 0.5 mL of blood was collected in sodium heparin tubes for hematological analysis. After blood collection, mice were euthanized, and major organs (heart, liver, spleen, lungs, kidneys, brain) were fixed in 4% paraformaldehyde for 48 h, embedded in paraffin, sectioned (4 μm), and stained with hematoxylin and eosin (H&E). Histopathological changes were observed under a light microscope.

### 2.21 Statistical Analysis

All experiments were performed in triplicate, and data are expressed as mean ± standard deviation (SD). Statistical significance was analyzed using SPSS 19.0 (IBM Corp., Armonk, NY, USA). One-way ANOVA followed by Tukey’s post hoc test was used to determine differences among groups.

## 3 Results and Discussion

### 3.1 Preparation and Characterization of hMLV

FLPs were prepared using the thin-film dispersion method.^32,40^ Based on preliminary experiments, single-factor optimization was performed to determine the formulation and process parameters. The optimized FLPsdisplayed uniform morphology and a zeta potential sufficient to maintain system stability. Characterization confirmed that the physicochemical properties of the prepared FLPs were satisfactory. Reparative M2 macrophages were generated following established protocols and validated by positive expression of the classical markers Arginase-1 (Arg-1) and CD206, proteins linked to immunoregulation, inflammation suppression, and tissue repair.^48^

M2 macrophage–derived extracellular nanovesicles (EVs) were isolated by differential centrifugation (see Figure 1). Activated M2 EVs were confirmed to express the exosomal marker CD81, verifying successful preparation of reparative M2 exosomes. No corresponding bands were observed in the FLPs core, indirectly demonstrating physical fusion between FLPs and M2 EVs. The morphology and particle size distribution of hMLV were characterized by transmission electron microscopy (TEM) and dynamic light scattering, revealing uniform nanostructures with an average diameter of 122 ± 5.4 nm (Figure 2A–D). The encapsulation efficiency (EE) and drug loading capacity of Fer-1 in hMLV were determined using an ultrafiltration centrifugation method. Results from three independent experiments showed an average EE of 92.17% and an average drug loading of 3.9 ± 1.21%. These data indicate that hMLV exhibits high encapsulation efficiency and favorable drug-loading capacity, supporting its potential for efficient delivery and synergistic therapy in subsequent experiments.In vitro release studies showed a minor burst release of Fer-1 from FLPs within 0–4 h, whereas the hMLV group exhibited sustained and gradual release, with a cumulative release of <70% at 36 h. The drug release profile of hMLV was significantly slower compared to that of FLPs, primarily attributed to its unique biomimetic hybrid structure. hMLV consists of a Fer-1-loaded liposomal core fused with an exosome membrane derived from M2 macrophages. This exosomal membrane is enriched in cholesterol, sphingomyelin, and various transmembrane proteins, forming a more compact and robust architecture that substantially enhances the overall stability and mechanical integrity of the nanovesicle, thereby restricting the diffusion rate of the encapsulated drug. Additionally, the hybrid membrane acts as a natural biological barrier, prolonging the diffusion pathway of Fer-1 from the inner core to the external environment, effectively minimizing burst release and enabling sustained, controlled release kinetics. This sustained release behavior not only prolongs systemic circulation time and enhances brain-targeted accumulation but also prevents premature drug leakage, offering prolonged and efficient therapeutic effects for traumatic brain injury.

**Figure 1:**
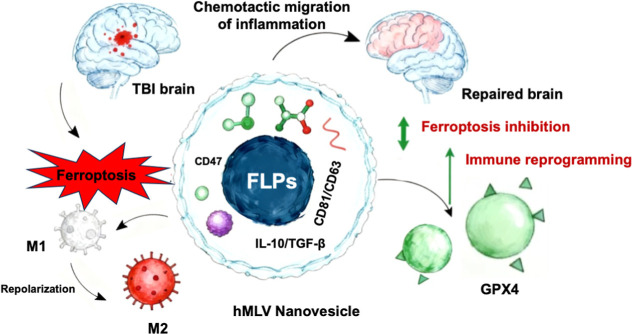
Mechanism of the hMLV-targeted delivery system. hMLV crosses the BBB via chemokine receptor-mediated transcytosis and accumulates at the injury site. Released Fer-1 inhibits ferroptosis by restoring GPX4 activity and reducing lipid peroxidation, thereby preventing neuronal death. Concurrently, exosome-derived IL-10 and TGF-β promote macrophage polarization toward the M2 phenotype, suppressing pro-inflammatory cytokine production. This dual action breaks the vicious cycle between oxidative stress, ferroptosis, and neuroinflammation, establishing a positive therapeutic feedback loop for brain repair.

**Figure 2:**
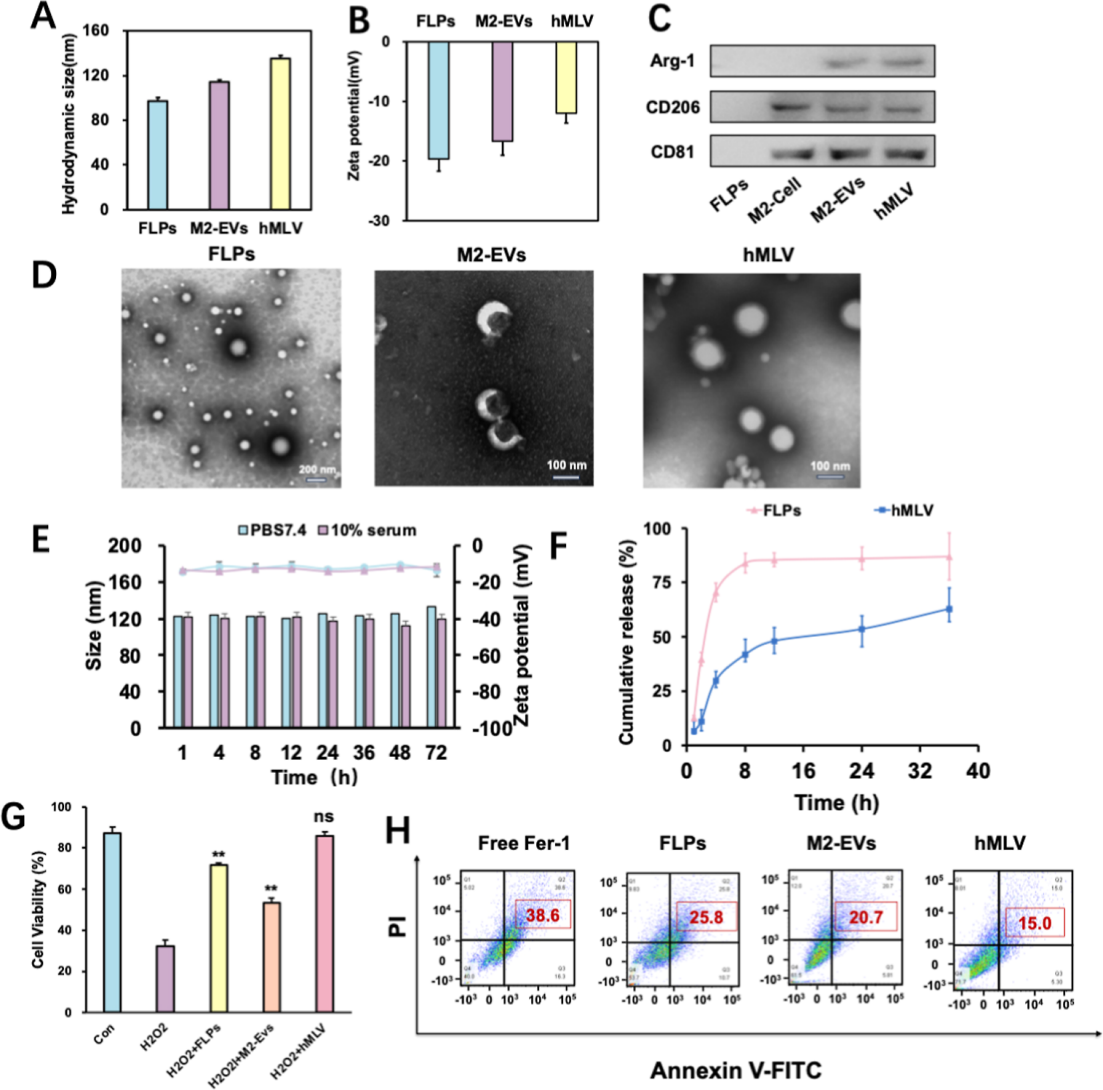
Preparation and characterization of hMLV. (A) Size distribution and zeta potential (B)­of different preparations (*n* = 3). (C) Western blotting of exosome-specific proteins in M2-EVs, FLPs, and hMLV (*n* = 3). (D) TEM images of different preparations. (E) In vitro drug release profiles in PBS (pH 7.4) with or without 10% serum (*n* = 3). (F) Serum stability of hMLV (*n* = 3). (G) Cell viability after oxidative stress with various formulations (*n* = 3). (H) Antiapoptosis results of H_2_O_2_-damaged HT22 cells before treatment with different treatment (*n* = 3). Data are presented as mean ± SD; *p < 0.05, **p < 0.01, ***p < 0.001; ns, not significant vs Con group.

Stability is a prerequisite for in vivo application. As shown in Figure 2E, hMLV remained stable in PBS containing 10% fetal bovine serum (FBS) for up to 72 h, with no appreciable change in hydrodynamic diameter. This in vitro stability implies that fusion of M2 EVs with FLPs limits protein corona formation and thereby improves colloidal stability. Enhanced stability is expected to facilitate immune evasion and prolong systemic circulation.^49^ Collectively, these data support the suitability of hMLV as a drug delivery platform (Figure 2F).

### 3.2 Targeted Delivery of hMLV to the TBI Site

To investigate whether hMLV can effectively cross the blood–brain barrier (BBB) and selectively accumulate at brain injury sites, we systematically evaluated its performance at both cellular and animal levels. Using an established in vitro BBB model,^50,51^ we assessed the uptake of DiD-labeled hMLV by HT22 neuronal cells. The results revealed significant differences in cellular uptake of nanoparticles across treatment groups. Compared with the control group, the uptake of M2-EVs and hMLVs by HT22 cells was markedly enhanced (Figure 3B). Notably, this uptake was further increased under inflammatory conditions mimicking BBB disruption. These findings suggest that hMLV possesses the ability to traverse the inflamed BBB and deliver therapeutic agents specifically to injured brain regions. This observation is consistent with the results from flow cytometry analysis. In summary, the in vitro BBB injury model, which incorporates fMLP to induce inflammation, effectively recapitulates the key pathological features of BBB disruption following TBI, providing a reliable platform for evaluating the targeted transcytosis capability of hMLV under disease-relevant conditions. To evaluate the immune evasion capability of hMLV, we designed an in vitro phagocytosis assay using RAW264.7 macrophages. DiD-labeled hMLVs and control FLPs were incubated separately with RAW264.7 cells, and the extent of nanoparticle uptake was assessed by confocal laser scanning microscopy. The results showed that the mean fluorescence intensity in the hMLV group was significantly lower than that in the FLP group, indicating markedly reduced phagocytic uptake and suggesting enhanced “don’t eat me” signaling and superior immune evasion capacity.

**Figure 3:**
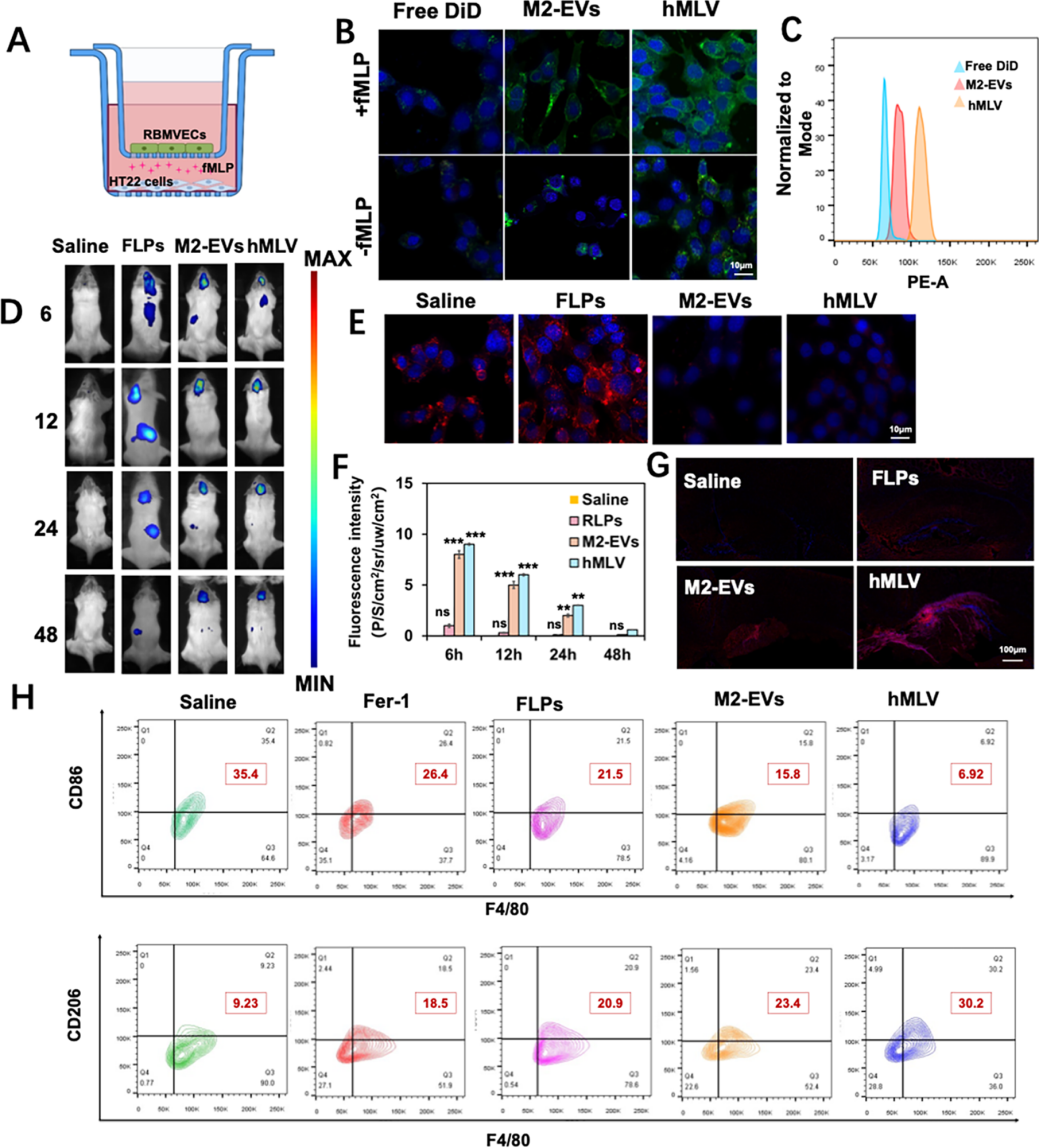
Targeted delivery of hMLV to the TBI site in vitro and in vivo. (A) Schematic diagram of the in vitro BBB model simulating the inflammatory state after TBI. (B) Representative CLSM images of hMLV penetration in the in vitro BBB model. (B) Quantitative analysis of BBB penetration in vitro. (C) Flow cytometric analysis of hMLV uptake efficiency in the BBB model (*n* = 3). (D) In vivo imaging of TBI mice following different treatments. (E) Cell uptake of hMLV by RAW264.7 cells (*n* = 3). (F) Statistical analysis of fluorescence content of hMLV in mice brains with imaging in vivo. (G) Immunofluorescence staining of hMLV in brain tissue after administration. (H) Representative flow cytometry plots of CD86^+^F4/80^+^ BV-2 cells in the different treatment groups (*n* = 3). Data are presented as mean ± SD; *p < 0.05, **p < 0.01, ***p < 0.001; ns, not significant vs Con group.

The biodistribution of hMLV in vivo was evaluated using a small-animal imaging system in a controlled cortical impact (CCI) mouse model. As shown in Figure 3D, the saline group exhibited minimal fluorescence, confirming that neither the CCI procedure nor saline interfered with DiR fluorescence. In the FLPs group, only weak cerebral fluorescence was detected, likely due to BBB disruption following injury that allowed partial penetration of lipid nanoparticles. By contrast, M2-EV and hMLV groups showed stronger cerebral fluorescence, attributable to the exosomal coating, which endowed the nanovesicles with chemotactic properties toward inflammatory sites. Among all groups, hMLV exhibited the strongest brain-targeting efficiency.

Furthermore, the fluorescent signal gradually accumulated in the cranial region over time and reached its peak at 24 h postadministration. To further validate the biodistribution profile, mice were euthanized at 24 h after injection, and major organs (brain, heart, liver, spleen, lungs, and kidneys) were harvested for ex vivo fluorescence imaging. The results showed that FLPs exhibited noticeable accumulation in the liver and spleen, consistent with the typical clearance pathway of nanoparticles via the mononuclear phagocyte system. In contrast, hMLV maintained significantly higher and more specific fluorescence intensity in the brain tissue, indicating superior blood–brain barrier penetration and brain-targeting capability. Notably, minimal fluorescence was detected in the liver of the hMLV group, suggesting a substantially reduced uptake by macrophages and implying enhanced immune evasion properties of this delivery system (Figure S1). These findings are consistent with the in vivo whole-body imaging data, further supporting the specific accumulation of hMLV at the TBI-affected sites.

Macrophage cells in the various treatment groups were also subjected to flow cytometry analysis to assess the expression of representative markers for the M1 phenotype (CD86) and the M2 phenotype (CD206). Following treatment with hMLV, the percentage of F4/80^+^CD86^+^ macrophage cells significantly decreased from 35.4% to 6.92% (Figures 3H, S2). In contrast, a notable increase in the percentage of F4/80^+^CD206^+^ cells was observed, rising from 9.23% to 30.2%. These findings indicated that hMLV could induce the polarization of macrophage cells from the proinflammatory M1 phenotype to the anti-inflammatory M2 phenotype Taken together, these results indicate that hMLV maintains favorable physiological activity and drug loading capacity in systemic circulation while exhibiting strong chemotaxis toward inflammatory cytokines in injured brain tissue, thereby ensuring efficient drug release at the target site.

### 3.3 The Neuroprotective Effect of hMLV after Traumatic Brain Injury

The therapeutic efficacy of hMLV against traumatic brain injury (TBI) was evaluated both in vitro and in vivo. To mimic the pathological environment of TBI, particularly oxidative stress and ferroptosis, an HT22 neuronal injury model was established by cotreatment with hydrogen peroxide and a ferroptosis inducer.^44^ The protective effect of hMLV on damaged HT22 cells was then assessed using the CCK-8 assay (Figure 2G). Compared with the control group, cell viability was significantly increased in the FLP, M2-EV, and hMLV groups, with the highest viability observed in the hMLV group. This enhanced protection can be attributed to two factors: (i) the sustained release of Fer-1 from hMLV, which effectively inhibited ferroptosis in HT22 cells, and (ii) the improved cellular uptake efficiency conferred by the exosomal coating, overcoming the limited uptake of FLPs. Flow cytometry further demonstrated that the apoptosis rate in the hMLV group (15.0%) was lower than that in the FLPs (25.8%) and M2-EV (20.7%) groups (Figure 4B). Collectively, these results indicate that hMLV effectively suppresses both ferroptosis and apoptosis in neuronal cells, thereby conferring robust neuroprotection.

**Figure 4:**
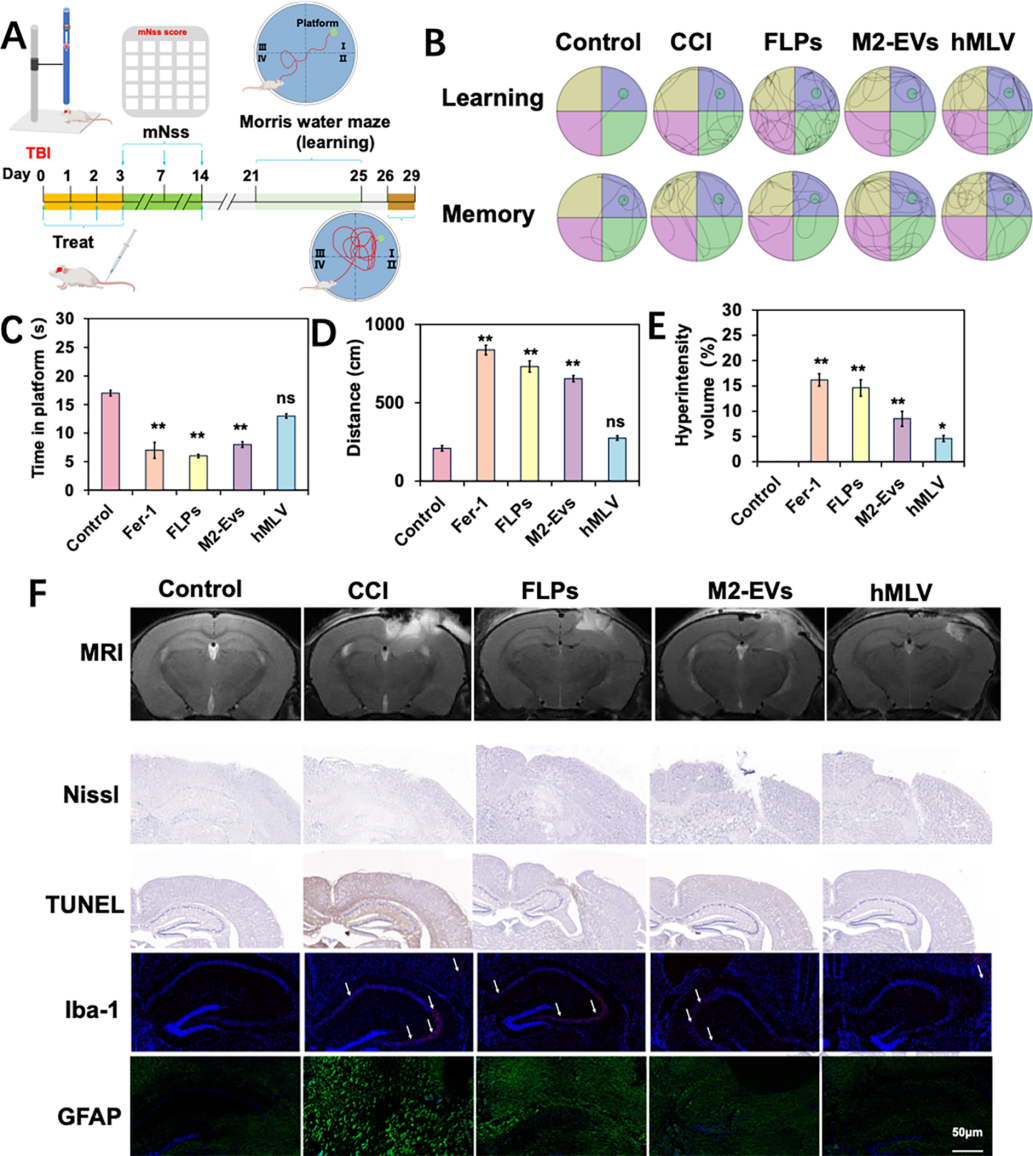
Neuroprotective effects of hMLV after TBI. (A) Schematic diagram of TBI model experiments. (B) Representative swimming trajectories during the learning and memory phases in the Morris water maze (*n* = 6). (C) During the memory experiment, searching time for the platform and swimming distance to the platform (D) (*n* = 6). (E) Quantification of the hyperintensity volume around the injured tissue (*n* = 6). (F) MRI images, IHC and IF staining showing hyperintensity volune, Nissl-positive neurons, apoptotic cells, Iba-1-positive microglia, and GFAP-positive astrocytes in the injured cortex of TBI mice across different treatment groups. Data are presented as mean ± SD *p < 0.05, **p < 0.01, ***p < 0.001; ns, not significant vs Con group.

The in vivo anti-TBI efficacy of the hMLV targeted delivery system was evaluated using the mouse CCI model. Neurological function was assessed in each group using the modified neurological severity score (mNSS), which evaluates motor, sensory, and balance abilities through multiple test components. As shown in Figure S3, the CCI group had significantly higher mNSS scores than the sham group, confirming successful model establishment. Seven days postinjury, the treatment groups exhibited significantly lower scores compared with the CCI group, indicating that the hMLV targeted delivery system exerted a therapeutic effect in TBI mice. Cognitive performance was further examined using the Morris water maze test. As shown in Figure 4C,D, CCI mice displayed significant learning impairments compared with the control group. Treatment improved spatial learning in all groups, while mice treated with hMLV exhibited the greatest recovery, as evidenced by more platform crossings and increased time and distance spent in the target quadrant. Additionally, the extent of brain edema in different treatment groups was further evaluated in vivo using magnetic resonance imaging (MRI). A significant increase in right hemispheric brain hyperintensity volume was observed in the injured group compared to the control group (Figure 4E,F). Together, these findings indicate that hMLV enhances both neurological and cognitive recovery following TBI.

All mice were sacrificed 1 week after drug administration, and brain tissues were collected for Nissl staining to assess neuronal integrity (Figure 4E). In the control group, neurons displayed abundant, dark-blue Nissl bodies, indicative of active protein synthesis. In contrast, the CCI group exhibited a significant reduction or even disappearance of Nissl bodies in the injured region, accompanied by gliosis and neuronal disruption, with some neurons exhibiting ruptured Nissl bodies. Treatment with FLPs, M2-EVs, or hMLVs preserved more Nissl bodies compared with the CCI group, with the hMLV group showing the greatest preservation. These results suggest that hMLV mitigates neuronal damage after TBI and helps maintain Nissl body integrity.

Brain tissue was also examined by TUNEL staining, performed using the avidin–biotin system, in which Streptavidin–HRP was conjugated to Biotin-dUTP, and DAB served as the HRP substrate to generate a brown precipitate. Hematoxylin was used for nuclear counterstaining, with DAB-positive apoptotic nuclei appearing brownish-yellow. In the control group, neuronal morphology was preserved, nuclei were clearly stained, and only minimal apoptosis was observed. By contrast, the CCI group exhibited a markedly higher proportion of TUNEL-positive cells, indicating extensive apoptosis. Treatment with the hMLV delivery system reduced the proportion of apoptotic cells compared with the CCI group, further supporting its neuroprotective effect.

To assess neuroinflammation status in brain tissue following TBI, we assessed the expression of glial fibrillary acidic protein (GFAP) and ionized calcium-binding adapter molecule-1 (Iba-1), which are established markers of astrocyte and microglial activation, respectively.^52^ After brain injury, astrocytes and microglia are rapidly activated, characterized by cellular swelling and branching. Microglia, the resident immune cells of the central nervous system, are responsible for sensing, maintenance, and defense, but their dysfunction in neurodegenerative diseases can exacerbate neuronal injury. Likewise, activated astrocytes release large amounts of inflammatory mediators. Therefore, immunofluorescence staining of GFAP and Iba-1 was performed to evaluate the extent of neuroinflammation after TBI (Figure 4E).

The hippocampus, which plays a critical role in learning and spatial memory, is particularly susceptible to TBI, and its degeneration is closely associated with poor prognosis. On day 7 post-TBI, the number of GFAP- and Iba-1-positive cells in the hippocampus was markedly increased, indicating enhanced astrocyte and microglial activation. Treatment with hMLV significantly reduced the number of GFAP- and Iba-1-positive cells compared with the untreated TBI group. These findings suggest that hMLV attenuates neuroinflammation in the hippocampus and thereby promotes neuronal repair.

### 3.4 Mechanism of hMLV on Neural Function Recovery after TBI In Vitro and In Vivo

To elucidate the mechanisms underlying the synergistic effect of ferroptosis-based immunotherapy in mitigating secondary brain injury after TBI, we first examined the inhibitory effect of hMLV on ferroptosis in neuronal cells both in vivo and in vitro. DCFH-DA staining was used to detect intracellular reactive oxygen species, a key trigger of ferroptosis.^53^ Confocal laser scanning microscopy images of HT22 cells treated with DMEM (control), FLPs, M2-EVs, or hMLV revealed strong green fluorescence in the control group, indicating elevated ROS levels (Figure 5A). In contrast, ROS fluorescence was markedly reduced in the FLPs and hMLV groups, with hMLV achieving greater suppression due to enhanced cellular uptake.

**Figure 5:**
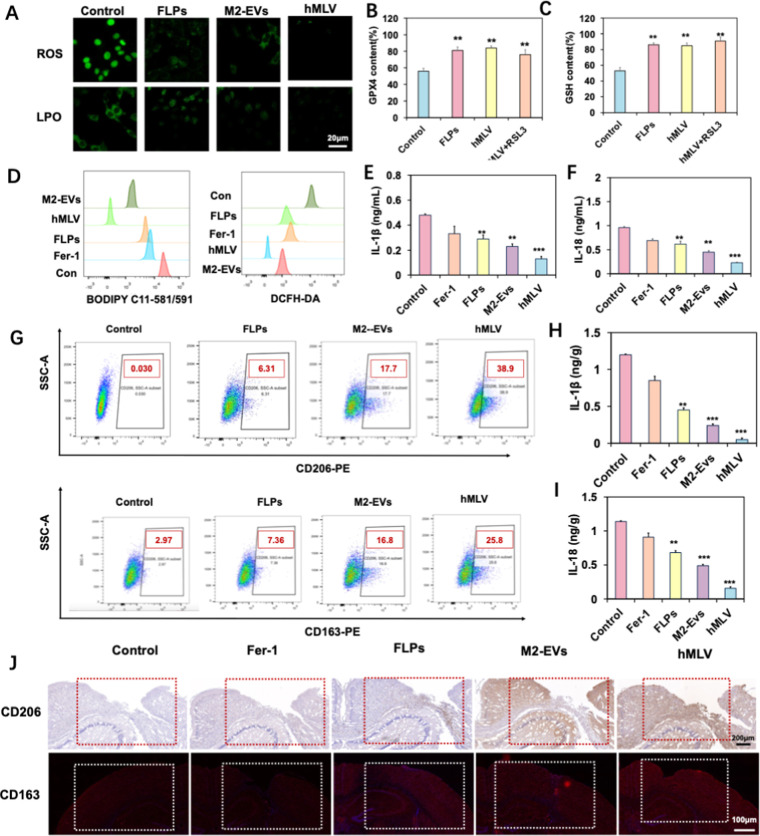
Mechanisms of hMLV in promoting neural function recovery after TBI in vitro and in vivo. (A) CLSM images of intracellular ROS and LPO in HT22 cells (*n* = 3). (B,C) Relative levels of GPX4 and GSH in HT22 cells after different treatments (*n* = 6). (D) Fluorescence images of brain tissues stained with DCFH-DA and BODIPY C11–581/591. (E,F) Levels of IL-1β and IL-18 in injured HT22 cells in vitro (*n* = 6). (G) Flow cytometry analysis of CD206-and CD163-positive M2 macrophages after hMLV treatment (*n* = 3). (H,I) Levels of IL-1β and IL-18 in vivo (*n* = 6). (J) Expression of CD206-and CD163-positive macrophages in the injured brain regions of TBI mice detected by IHC and IF staining. Data are presented as mean ± SD *p < 0.05, **p < 0.01, ***p < 0.001; ns, not significant vs Con group.

To further assess lipid peroxidation, HT22 cells were stained with BODIPY C11–581/591 and analyzed by CLSM. Compared with other groups, hMLV-treated cells exhibited significantly lower levels of lipid peroxides (LPO), indicating potent inhibition of ferroptosis. Together, these findings demonstrate that hMLV effectively reduces ROS accumulation and lipid peroxidation, thereby contributing to neuronal protection and repair after TBI. GPX4 is a key regulator of the ferroptosis pathway, with reduced glutathione (GSH) serving as its cofactor. Depletion of GSH inactivates GPX4, leading to the accumulation of lipid peroxides and their degradation products. Thus, promoting GPX4 activity is an effective strategy to inhibit ferroptosis. To investigate the effects of hMLV on ferroptosis regulation, we quantified GPX4 and GSH levels in HT22 cells using ELISA.^54,55^ Compared with the control and M2-EV groups, GPX4 levels were significantly elevated in cells treated with FLPs or hMLV (Figure 5B, C). However, cotreatment with RSL3, a GPX4 inhibitor, markedly reduced GPX4 expression in hMLV-treated cells. Similarly, intracellular GSH levels were significantly higher in the hMLV group compared with the control and M2-EVs groups (Figure 5C). These results indicate that hMLV maintains GPX4 activity and intracellular GSH levels, thereby suppressing ferroptosis in neuronal cells.

We further examined the in vivo mechanism of hMLV using the CCI model. Fluorescence cytometry images of brain tissue stained with BODIPY C11–581/591 and DCFH-DA revealed that hMLV treatment markedly LPO and ROS accumulation compared with FLPs and NK-EVs (Figure 5D). This reduction was consistent with in vitro findings and reflects the targeted delivery of Fer-1 by hMLV to the injured brain. Moreover, brain tissues from hMLV-treated mice exhibited significantly higher GPX4 and GSH levels than those from other treatment groups. Collectively, these data suggest that hMLV releases Fer-1 to inhibit ROS production and lipid peroxidation, thereby maintaining GPX4 activity and GSH levels, which contributes to its neuroprotective effect after TBI.

Inflammatory responses are positively correlated with the severity of TBI.^56^ Following TBI, the expression of pro-inflammatory cytokines, including interleukin-18 (IL-18) and interleukin-1β (IL-1β), was markedly upregulated, further exacerbating neuroinflammation (Figure 5E,F). Continuous treatment with hMLV formulations reduced IL-18 and IL-1β levels to varying degrees and alleviated TBI-associated symptoms. These results indicate that hMLV effectively attenuates neuroinflammation and protects against delayed neurodegenerative changes. The therapeutic effect is achieved through the combined actions of ferroptosis inhibition and immunomodulation, while minimizing adverse effects on healthy tissues, highlighting the potential of hMLV for optimizing TBI treatment.

We further assessed macrophage polarization in vivo. Specifically, we focused on M2 macrophage polarization, which plays a central role in regulating excessive inflammatory responses and promoting neuronal repair.^57,58^ Flow cytometry of brain tissue demonstrated that, compared with the control group, the numbers of CD163 positive and CD206 positive macrophages were markedly elevated in the hMLV- and M2-EVs–treated groups, whereas only modest increases were observed in the FLPs group (Figure 5G). This is also consistent with the results of IHC staining (Figure 5J). Moreover, the levels of inflammatory factors in brain tissue were significantly reduced. All of these findings indicate that hMLV retains anti-inflammatory components from M2-EVs during fusion, thereby enhancing its ability to modulate macrophage polarization and contributing to its neuroprotective effect.^59^

Notably, the therapeutic effect observed in this study is not attributable to isolated intervention on a single pathway, but rather results from coordinated modulation of multiple critical nodes within the pathological network of traumatic brain injury (TBI). Specifically, oxidative stress serves as a central link connecting ferroptosis and neuroinflammation. In the early phase after trauma, substantial accumulation of reactive oxygen species (ROS) not only directly damages lipid components of cell membranes, but also depletes glutathione (GSH), leading to inhibition of GPX4 activity and initiation of ferroptosis. Upon undergoing ferroptosis, neurons release damage-associated molecular patterns (DAMPs), such as HMGB1, ATP, and lipid peroxidation products, which are recognized by microglia and infiltrating macrophages. This recognition triggers activation of the NLRP3 inflammasome pathway, promoting maturation and secretion of IL-1β and IL-18, thereby exacerbating neuroinflammatory responses. In turn, activated M1-type pro-inflammatory macrophages further amplify local oxidative stress through the release of nitric oxide (NO) and ROS, creating a self-amplifying pathogenic cascade.

The design of hMLV precisely targets key points within this detrimental cycle. On one hand, Fer-1 encapsulated in the core effectively scavenges lipid free radicals, restores GPX4 function, suppresses ferroptosis, and reduces DAMPs release. On the other hand, immunomodulatory cytokines such as IL-10 and TGF-β present on the exosomal membrane can reprogram macrophages toward an anti-inflammatory M2 phenotype, downregulate expression of pro-inflammatory factors, and reduce the production of ROS and NOS. The synergistic action of these two mechanisms not only disrupts the positive feedback loop among oxidative stress, ferroptosis, and inflammation, but also promotes restoration of homeostasis in the neural microenvironment.

### 3.5 In Vivo Compatibility of hMLV

An ideal nanocarrier should not only possess favorable physicochemical properties but also exhibit minimal toxicity and high biocompatibility.^60–62^ To preliminarily evaluate the safety of the hMLV drug delivery system, we performed histopathological, hematological, and biochemical analyses in mice following in vivo efficacy studies. Key organs, including the brain, heart, liver, spleen, lungs, and kidneys, were collected, paraffin-embedded, sectioned, and stained with hematoxylin and eosin (H&E) for microscopic examination (Figure 6). No significant pathological damage or abnormal changes were observed in hMLV-treated mice compared with the untreated group, suggesting the absence of obvious organ toxicity.

**Figure 6:**
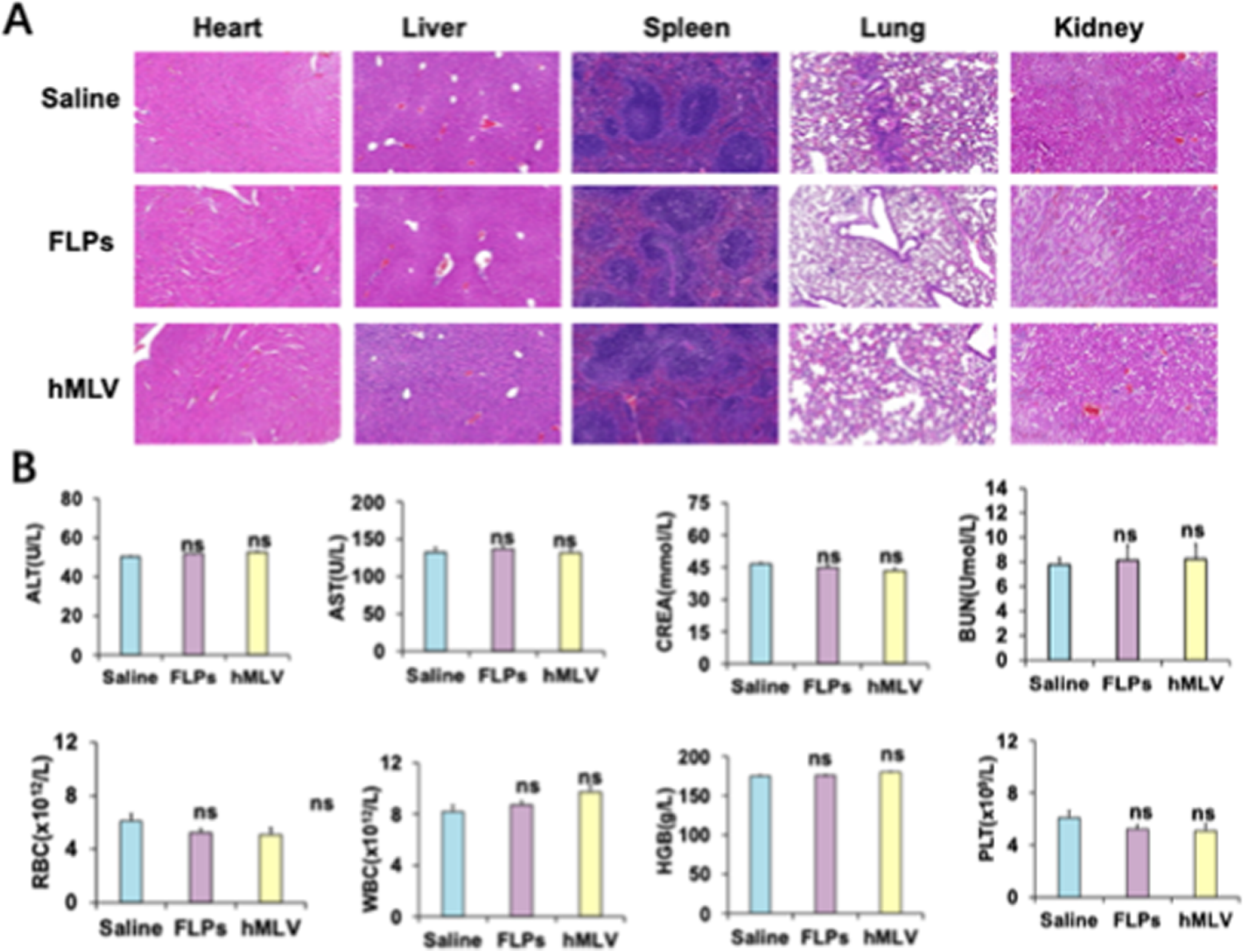
In vivo safety evaluation of hMLV nanosystems. (A) Representative H&E-stained sections of major organs from mice treated with different formulations. (B) Results of routine blood tests and liver/kidney function assays in treated mice (*n* = 4). Data are presented as mean ± SD *p < 0.05, **p < 0.01, ***p < 0.001; ns, not significant vs saline group.

In addition, routine blood tests and assessments of liver and kidney function were performed. All parameters remained within normal ranges, with no significant differences between hMLV-treated and untreated groups. Blood cell counts also showed no abnormalities. These results demonstrate that hMLV does not induce detectable toxicity in major organs, nor does it impair hematological or hepatic/renal function during in vivo application. The good biocompatibility and safety profile of hMLV support its potential for future clinical translation.

## 4 Conclusions

In this study, we developed a biomimetic hybrid nanovesicle (hMLV) system that synergistically targets ferroptosis and immune dysregulation in traumatic brain injury (TBI). By integrating Fer-1 with M2 macrophage-derived exosomes, hMLVs simultaneously inhibit neuronal ferroptosis and reprogram the immune microenvironment. Compared with single-component therapies, hMLVs provided superior neuroprotection and functional recovery in both in vitro and in vivo TBI models. hMLVs effectively penetrated the blood–brain barrier and localized to injured brain regions, where Fer-1 release inhibited ferroptosis by reducing lipid peroxidation and restoring glutathione peroxidase 4 (GPX4) activity. Concurrently, exosome-derived components promoted macrophage polarization toward a reparative M2 phenotype, thereby attenuating neuroinflammation. This dual mechanism establishes a self-reinforcing therapeutic cycle in which ferroptosis inhibition alleviates inflammatory triggers, while M2 polarization reduces oxidative stress. Our findings underscore the potential of hMLVs as a clinically translatable nanoplatform for secondary injury management following TBI. By simultaneously modulating ferroptosis and immune responses, hMLVs provide a comprehensive strategy that addresses the multifactorial pathophysiology of TBI. Future studies will optimize formulation parameters and evaluate long-term efficacy and safety in preclinical models, with the goal of advancing this approach toward clinical application. Beyond TBI, this work also highlights the broader promise of synergistic nanotherapies for neurodegenerative diseases.

## Supplementary Material



## Notes

Fluorescence imaging in brain and major tissues after different treatments; Quantitative analysis of CD86^+^F4/80^+^ and CD206^+^F4/80^+^ macrophage cells in the different treatment groups; Modified neurological severity scores (mNSS) assessed at 7 and 14 days post-TBI (PDF)
