# Supplementary material for: Co-Delivery of Ferrostatin‑1 and M2 Macrophage-Derived Exosomal Signals via Engineered Hybrid Nanovesicles Enables Synergistic Neuroprotection in Traumatic Brain Injury
Source: ACS Appl Mater Interfaces. 2026 Apr 7;18(15):21791–803. doi: 10.1021/acsami.6c01290 (PMC13107366; doi:10.1021/acsami.6c01290)
Supplement: Supplementary file 1 [file am6c01290_si_001.pdf]

Supporting Information

Co-delivery of Ferrostatin-1 and M2 macrophage-derived exosomal signals via engineered hybrid nanovesicles enables synergistic neuroprotection in traumatic brain injury

Wenyan Hao[a,b,c], Nan Sun[b], Ruifen Xue[a], Junkai Chang[d], Xiaocong Pang[a], Ying Zhou[a,c]*, Chunsheng Gao[b]**

[a]Department of Pharmacy, Peking University First Hospital, Xishiku Street, Xicheng District, Beijing100034, China, 100191 Beijing, China

[b]State Key Laboratory of Toxicology and Medical Countermeasures, Beijing Institute of Pharmacology and Toxicology, Beijing100085, China

[c]State Key Laboratory of Advanced Drug Delivery and Release Systems, Shandong Luye Pharmaceutical Co., Ltd., Yantai, Shandong 264003, PR China

[d]University College London, Flat 21, Dickens house, London, UK

** To whom correspondence should be addressed.*

Corresponding Authors

Xiaocong Pang: pangxiaocong1227@163.com

Ying Zhou: zhouying0321@126.com

Chunsheng Gao:gaocs@bmi.cn

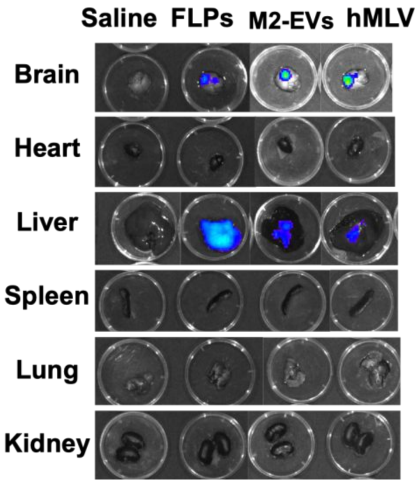

Figure S1. Fluorescence imaging in brain and major tissues after different treatments.

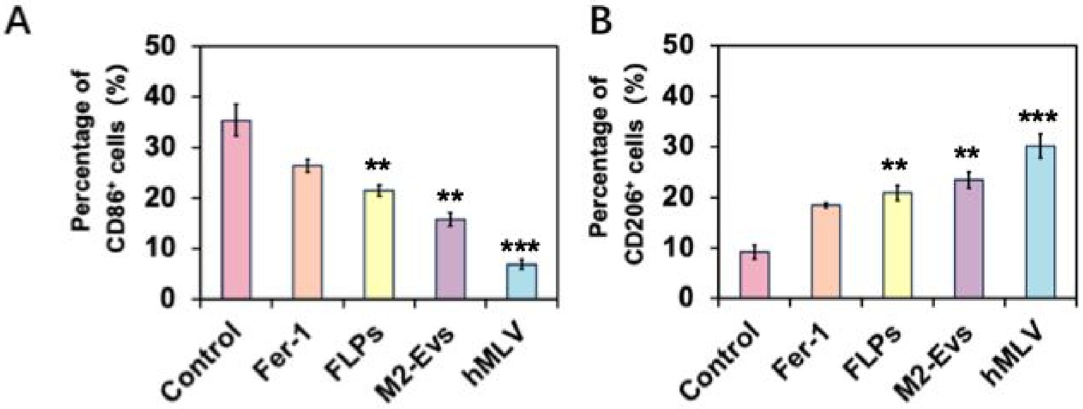

Figure S2. (A) Quantitative analysis of $CD86^{+}F4/80^{+}$and $CD206^{+}F4/80^{+}$ (B) macrophage cells in the different treatment groups. Data are presented as mean ± SD; $*p < 0.05$, $**p < 0.01$, $***p < 0.001$; ns, not significant vs. Con group.

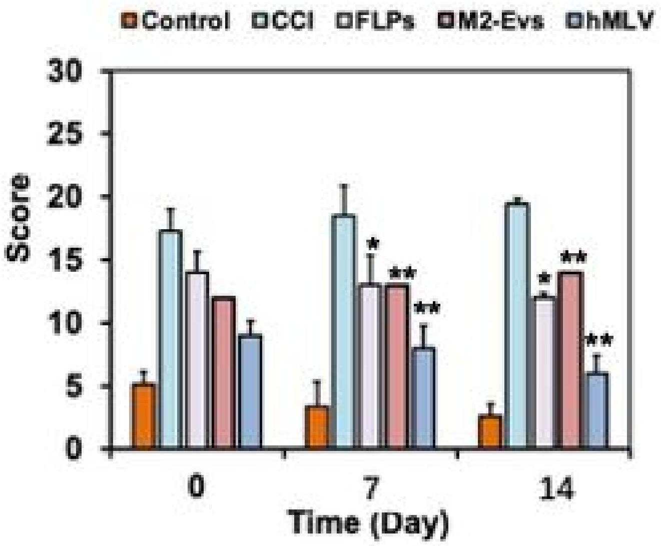

Figure S3. Modified neurological severity scores assessed post-TBI. Data are presented as mean ± SD; *$p < 0.05$, **$p < 0.01$, ***$p < 0.001$; ns, not significant vs. CCI group.
